# Detecting Interactions in High‐Dimensional Data Using Cross Leverage Scores

**DOI:** 10.1002/bimj.70014

**Published:** 2024-11-29

**Authors:** Sven Teschke, Katja Ickstadt, Alexander Munteanu

**Affiliations:** ^1^ Faculty of Statistics TU Dortmund University Dortmund Germany; ^2^ Lamarr‐Institute for Machine Learning and Artificial Intelligence Dortmund Germany

**Keywords:** cross leverage scores, genetics, high‐dimensional data, interaction effects, sketching, variable selection

## Abstract

We develop a variable selection method for interactions in regression models on large data in the context of genetics. The method is intended for investigating the influence of single‐nucleotide polymorphisms (SNPs) and their interactions on health outcomes, which is a p≫n problem. We introduce cross leverage scores (CLSs) to detect interactions of variables while maintaining interpretability. Using this method, it is not necessary to consider every possible interaction between variables individually, which would be very time‐consuming even for moderate amounts of variables. Instead, we calculate the CLS for each variable and obtain a measure of importance for this variable. Calculating the scores remains time‐consuming for large data sets. The key idea for scaling to large data is to divide the data into smaller random batches or consecutive windows of variables. This avoids complex and time‐consuming computations on high‐dimensional matrices by performing the computations only for small subsets of the data, which is less costly. We compare these methods to provable approximations of CLS based on sketching, which aims at summarizing data succinctly. In a simulation study, we show that the CLSs are directly linked to the importance of a variable in the sense of an interaction effect. We further show that the approximation approaches are appropriate for performing the calculations efficiently on arbitrarily large data while preserving the interaction detection effect of the CLS. This underlines their scalability to genome wide data. In addition, we evaluate the methods on real data from the HapMap project.

## 1 Introduction

In this paper, we present a method to quickly and efficiently detect and select interaction effects in large data sets. In addition to the main effects, interaction effects are often of great relevance, for instance, when detecting associations of interacting genetic variations with certain diseases or physical conditions (Li et al. 2014). We consider single‐nucleotide polymorphisms (SNPs), which are individual variations of nucleotides in the (human) DNA with a prevalence of more than 1% (in contrast, when the prevalence is less than 1%, it is called a mutation). Not only individual SNPs, but also interactions between SNPs can be associated with a certain disease such as breast cancer (Chuang et al. 2014) or venous thrombosis (Greliche et al. 2013). In general, it is difficult to search for important interaction effects, because all possible combinations of p variables have to be considered individually and have to be included in the model. In a model with p variables, the amount of possible two‐way combinations is already quadratic p2=Θ(p2) and the number of choices grows exponentially in the order of interactions k, as pkk≤pk≤epkk. Finally, the number of all possible combinations of any degree sum up to $2^{p}$. This poses a crucial limitation, as it is common in genetics to consider higher order interactions (Schwender et al. 2012). Even for relatively small p, it is prohibitive to consider all possible interactions and it becomes increasingly difficult in higher dimensional data sets, for instance, in genetics where data sets with several millions of variables but comparably small numbers of observations n≪p are common. Nevertheless, research on this topic is very difficult because most available statistical methods fail in these extremely high‐dimensional settings. Prominent examples include logic regression (Ruczinski, Kooperberg, and LeBlanc 2003) or the maximum entropy conditional probability model (MECPM) approach (Miller et al. 2009), which are limited to a small number of variables.

In this paper, we introduce a method for calculating the so‐called cross leverage scores (CLSs) that indicate for each variable their leverage on the outcome variable and in particular their participation in an interaction effect. In particular, we avoid considering every possible combination of variables. By considering the original matrix of variables as a whole, we only need to calculate p individual scores in total. These indicate whether a variable is important or not in terms of an interaction effect. The method is therefore characterized by the fact that we consider the full multivariate model instead of p univariate models in which ones checks whether a single variable has an effect or not, which has so far been common (Uffelmann et al. 2021) but is neither desirable nor sustainable from a statistical point of view.

Indeed, any multivariate information on interaction effects is lost in single‐variable analyses. In particular, it can occur that the variables participating in important SNP interaction effects have only moderate or no influence at all if their individual main effect is considered (Li et al. 2014). Another issue is that several works claim genome‐wide studies but consider only a few thousand variables (e.g., Terada et al. 2016; Yang et al. 2008). Such methods often do not scale to arbitrarily large data without further computational improvements.

Methods that perform a hierarchical search for interactions are common, for example, the genome‐wide association analysis using LASSO‐penalized logistic regression (Wu et al. 2009). An example for a two‐step hierarchical search first selects only a small subset of variables with large main effects and in a second step searches for interacting variables only within this subset. A variable that interacts with others, but is missing an individual main effect would thus be removed from the consideration before the actual interaction analysis. Here, we propose a new hierarchical approach: in a first step, a quick variable selection is performed to reduce the number of variables based on CLS, such that variables participating in an interaction are retained. This enables in a second step to apply a more sophisticated method such as logic regression (Ruczinski, Kooperberg, and LeBlanc 2003), which is not readily applicable for high‐dimensional data sets, but good in finding interaction effects in scenarios with smaller p.

The idea of using CLS for this purpose was first mentioned in previous work of our group (Ding, Ickstadt, and Munteanu 2022; Parry et al. 2021). We build on this idea and further develop the approach of using CLS as a tool for detecting variable interactions. Here, we make this approach available for genome‐wide studies by developing scalable approximations with small bounded error guarantees, and applicable to arbitrarily large data sets. The approximation methods can also be extended to a pure data stream algorithm that reads the data successively while using only very limited memory of the computing environment such as R (for example, in the context of genetics, chromosome by chromosome).

Our paper is structured as follows. First, we will motivate the use of CLS for detecting interactions and selecting the participating variables. Then, we will show how they can be suitably approximated so that the method becomes applicable to very large data sets. In a simulation study, we will analyze how well the method is able to detect important variables participating in interactions of different order, and due to certain similarities, we compare their performance with correlations and uniform sampling as a baseline method. In particular, we will also give an example, where it is impossible to detect important interactions using correlations, but our CLSs reveal the participating variables. In addition, we will evaluate the effect of variable preselection by our and other methods in a simulated and a real data scenario.

## 2 Motivation

**FIGURE 1:**
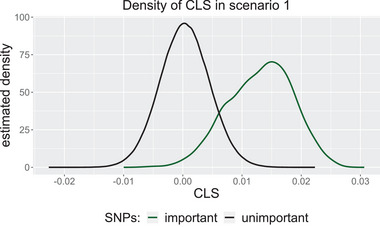
Kernel density estimates of the cross leverage scores of the important (green) and unimportant (black) variables.

To show that the CLSs are a useful measure for distinguishing between important and unimportant variables, we consider a scenario in which we simulate n=120 observations with one single important two‐way interaction and 1998 noisy variables. Note that n≪p. In total, we simulate 1000 data sets independently in the same manner. The simulations are described in detail in Section 4.1. We calculate the CLS between the individual variables and the binary target variable y as formally described in Section 3. In Figure 1, we plot the kernel density estimates of the calculated scores over all data sets, distinguishing between unimportant and important variables by color.

We see that the kernel density estimates for the unimportant and important variables differ strongly. The CLSs of the unimportant noise variables are concentrated around zero, while the CLSs of the important variables are larger. This suggests that we can use the CLS as a measure for distinguishing between unimportant and important variables and that we should select the variables with the largest CLS.

We will see later in Proposition 3.1 that each CLS equals their corresponding parameter in a least squares solution up to a small bounded additive error. This might suggest that it is just a different way of detecting main effects. We thus show that we actually measured the interaction effect and not solely main effects. To this end, we simulate a scenario in which the main effects of the first two variables are chosen to be negligibly small and no interaction effect is present. For a direct comparison, we consider the same scenario but with an added high interaction effect for the same two variables.

**FIGURE 2:**
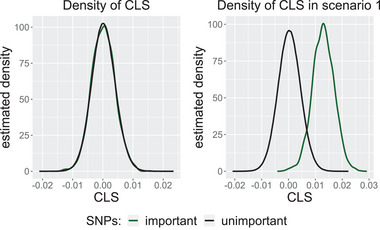
Kernel density estimates of the cross leverage scores of the important (green) and unimportant (black) variables. On the left, we consider two main effects with marginal influence, and in the right plot, we add a high interaction effect while the main effects persist to be low.

In the left plot in Figure 2, we see that the CLS of the first two variables cannot be distinguished from the other noise variables due to the absence of main or interaction effects. In contrast, we see in the right plot that we can distinguish between the important and unimportant variables using CLS because they indicate that the two variables participate in the added interaction. Although main effects are not increased in the generating model, they show up in the fitted model.

Using a toy example (Parry et al. 2021), we show that a variable selection with CLS outperforms the selection with correlation. We construct a data set with n=16 observations and p=60 variables with $x_{ij}\in \left\{0,1\right\}$ for i∈{1,…,n} and j∈{1,…,p}, and a binary response y. We construct the data set in such a way that the response takes the value y=1 whenever $x_{1}=x_{2}=1$ or $x_{3}=x_{4}=1$, and y=0 otherwise. By construction, there are two two‐way interactions. The remaining values for $x_{5}$ to $x_{60}$ are chosen from {0,1} uniformly at random:

(1) $$X=\left[\begin{array}{ccccccc} 1 & 1 & 0 & 1 & x_{1,5} & \cdots & x_{1,60}\\ 1 & 1 & 1 & 0 & x_{2,5} & \cdots & x_{2,60}\\ 0 & 0 & 1 & 1 & x_{3,5} & \cdots & x_{3,60}\\ 0 & 0 & 1 & 1 & x_{4,5} & \cdots & x_{4,60}\\ 1 & 0 & 0 & 1 & x_{5,5} & \cdots & x_{5,60}\\ 1 & 0 & 1 & 0 & x_{6,5} & \cdots & x_{6,60}\\ 0 & 1 & 0 & 1 & x_{7,5} & \cdots & x_{7,60}\\ 0 & 1 & 1 & 0 & x_{8,5} & \cdots & x_{8,60}\\ 1 & 1 & 0 & 1 & x_{9,5} & \cdots & x_{9,60}\\ 1 & 1 & 1 & 0 & x_{10,5} & \cdots & x_{10,60}\\ 0 & 0 & 1 & 1 & x_{11,5} & \cdots & x_{11,60}\\ 0 & 0 & 1 & 1 & x_{12,5} & \cdots & x_{12,60}\\ 1 & 0 & 0 & 1 & x_{13,5} & \cdots & x_{13,60}\\ 1 & 0 & 1 & 0 & x_{14,5} & \cdots & x_{14,60}\\ 0 & 1 & 0 & 1 & x_{15,5} & \cdots & x_{15,60}\\ 0 & 1 & 1 & 0 & x_{16,5} & \cdots & x_{16,60} \end{array}\right],y=\left[\begin{array}{c} 1\\ 1\\ 1\\ 1\\ 0\\ 0\\ 0\\ 0\\ 1\\ 1\\ 1\\ 1\\ 0\\ 0\\ 0\\ 0 \end{array}\right].$$

The data, especially the first two variables, are constructed in such a way that $\text{cor}\left(x_{1},y\right)=\text{cor}\left(x_{2},y\right)=0$. So, when selecting the variables according to their correlation with the response, we would never select the first two variables $x_{1}$ and $x_{2}$. In contrast, the CLSs of the first two variables are nonzero. It is therefore conceivable that we detect them being part of the two‐way interaction by selection via CLS. Of course, this is no guarantee, since the CLS may also depend on the other (noisy) variables.

**FIGURE A1:**
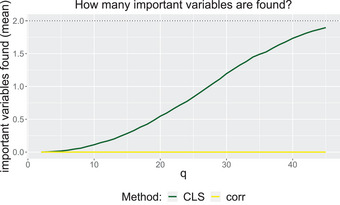
Toy example: On the *y*‐axis, we plot how many of the first two important variables we found on average when we choose the variables with the q largest cross leverage scores (green), respectively, largest correlation (yellow).

We thus conduct a small simulation study in which we simulate 1000 data sets according to the scheme described above in Equation (1). We count how often how many of the first two important variables are detected when selecting the variables with the q=⌈nlogn⌉=45 largest CLS or correlation. In the case of CLS, we find two out of two important variables in the median average, and zero out of two in the case of correlation. Consider Figure A1 in Appendix A.2 to see that even for small q where the selection with CLS deteriorates, it still outperforms the selection using correlations. This example shows that in some cases, we can detect interaction effects with CLS, where correlations necessarily miss the relevant information and thus miss the important variables.

## 3 Methods

As mentioned before, the idea to consider the CLSs is based on a paper of Parry et al. (2021). Since we deal with a p≫n problem, we exchange the role of observations and variables: to make our notation consistent with previous literature, where the case n≫p was treated (Drineas et al. 2012), we consider the matrix

(2) $$\tilde{X}={\left[X,y\right]}^{T}\in R^{\tilde{p}\times n}$$

with $\tilde{p}=p+1$ and p the number of variables and n the number of observations. $X\in R^{n\times p}$ is the data matrix and $y\in R^{n}$ denotes the response. We obtain the CLS from the off‐diagonal entries of the hat matrix H of $\tilde{X}$. The hat matrix H is given by $H=QQ^{T}$ (Hoaglin and Welsch 1978) where Q forms an orthonormal basis for the column space of $\tilde{X}$, which can be obtained by its QR‐decomposition $\tilde{X}=QR$ (Golub and Van Loan 1996). Since we are only interested in the CLS $c_{i\tilde{p}}$ between the variables i∈{1,…,p} and the response y, we can avoid the time‐consuming matrix multiplication of $Q\in R^{\tilde{p}\times n}$ and $Q^{T}\in R^{n\times \tilde{p}}$ by instead calculating only the dot products of rows $Q_{i\cdot }$ with row $Q_{\tilde{p}\cdot }$:

(3) $$c_{j\tilde{p}}=\left\langle Q_{i\cdot },Q_{\tilde{p}\cdot }\right\rangle ,\;i\in \left\{1,\text{…},p\right\}.$$

Equation (3) is computed from the orthogonal basis Q. This means that the CLSs correspond to the leverage of individual variables on the correlation of the multidimensional subspace spanned by the data with the outcome variable, rather than correlations of single variables. Our intuition is that an according subselection thus carries more information on the multivariate structure, and is capable of retaining interaction effects as shown in our previous example in Figure 2.

Under the mild assumption that the subspace is well aligned with the response vector, we show next that each CLS $c_{i,p+1}$ for i∈[p] equals their corresponding parameter in the least squares solution up to a small additive error. We note that variables with large parameters are thus recovered by CLS, and are more likely to participate in an interaction, since parameters near zero would cancel a possible interaction effect.

Proposition 3.1 Let $\tilde{X}=\left[X,y\right]\in R^{n\times (p+1)}$, for p≫n, where X and thus also $\tilde{X}$ have full rank n. Let $\tilde{X}=U\Sigma V^{T}$ be its SVD. Consider the smallest norm solution to the $\ell_{2}$ regression problem: $\beta^{OLS}\in \arg\min_{\beta \in R^{p}}{∥X\beta -y∥}_{2}^{2}\;$. Assume for $\frac{1}{2}>\eta >0$ that $\sum_{i=1}^{p}{\langle V_{i\cdot },V_{y\cdot }\rangle }^{2}\geq \frac{1}{\eta }{∥V_{y}∥}_{2}^{4}$. Then, it holds that

$$\begin{array}{c} \max_{i\in [p]}\left|\beta_{i}^{OLS}-c_{i,p+1}\right|<\frac{3\eta }{2}. \end{array}$$

See Appendix A.1.□

### 3.1 CLS Calculation

The obvious bottleneck of the approach described above is the QR‐decomposition with running time $\Theta (pn^{2})$ (Golub and Van Loan 1996), which is prohibitively slow to compute for p as large as several millions. The data might not even fit into main memory, which aggravates the situation. However, without the Q matrix, we are not able to calculate the CLS. Massively parallel QR‐decomposition algorithms are applicable in such cases but this requires a large compute cluster, which is not always available and this is no remedy for the total workload that remains $\Theta (pn^{2})$ (Demmel et al. 2012).

In this paper, we describe and compare three possible solutions to the problem that easily run on standard commodity hardware: specifically, we introduce two new heuristics, the *Sliding Window approach* and the *Random Window approach*. In both cases, instead of one QR‐decomposition for a large matrix, a lot of QR‐decompositions for many small submatrices are performed and their results are merged in a suitable way, akin to the Merge & Reduce technique (Geppert et al. 2020). The third solution is the *Sketching approach* based on the ideas of Drineas et al. (2012). The QR‐decomposition is calculated after reducing the large dimension $\tilde{p}$ by sketching $\tilde{X}\in R^{\tilde{p}\times n}$ to obtain ${\tilde{X}}_{★}\in R^{r\times n}$ with significantly smaller dimension $r\ll \tilde{p}$.

In what follows, the main goal is to approximate the CLS in an efficient way, based on which we make a preselection retaining the important variables. This enables more refined methods that are not suitable for large data sets—such as logic regression (Ruczinski, Kooperberg, and LeBlanc 2003)—to operate on the reduced data subsequently. We select the variables that have the most extreme CLS. It was recommended to select q=⌈nlogn⌉ variables in (Parry et al. 2021), which is confirmed in our experimental findings. Theoretically, this is supported by the coupon collector's problem (Erdős and Rényi 1961) that requires oversampling by a logn factor so that the selection contains at least as many variables to ensure that the submatrix preserves the full rank n of the original matrix (Tropp 2011).

### 3.2 New Window‐Based Approaches

**FIGURE 3:**
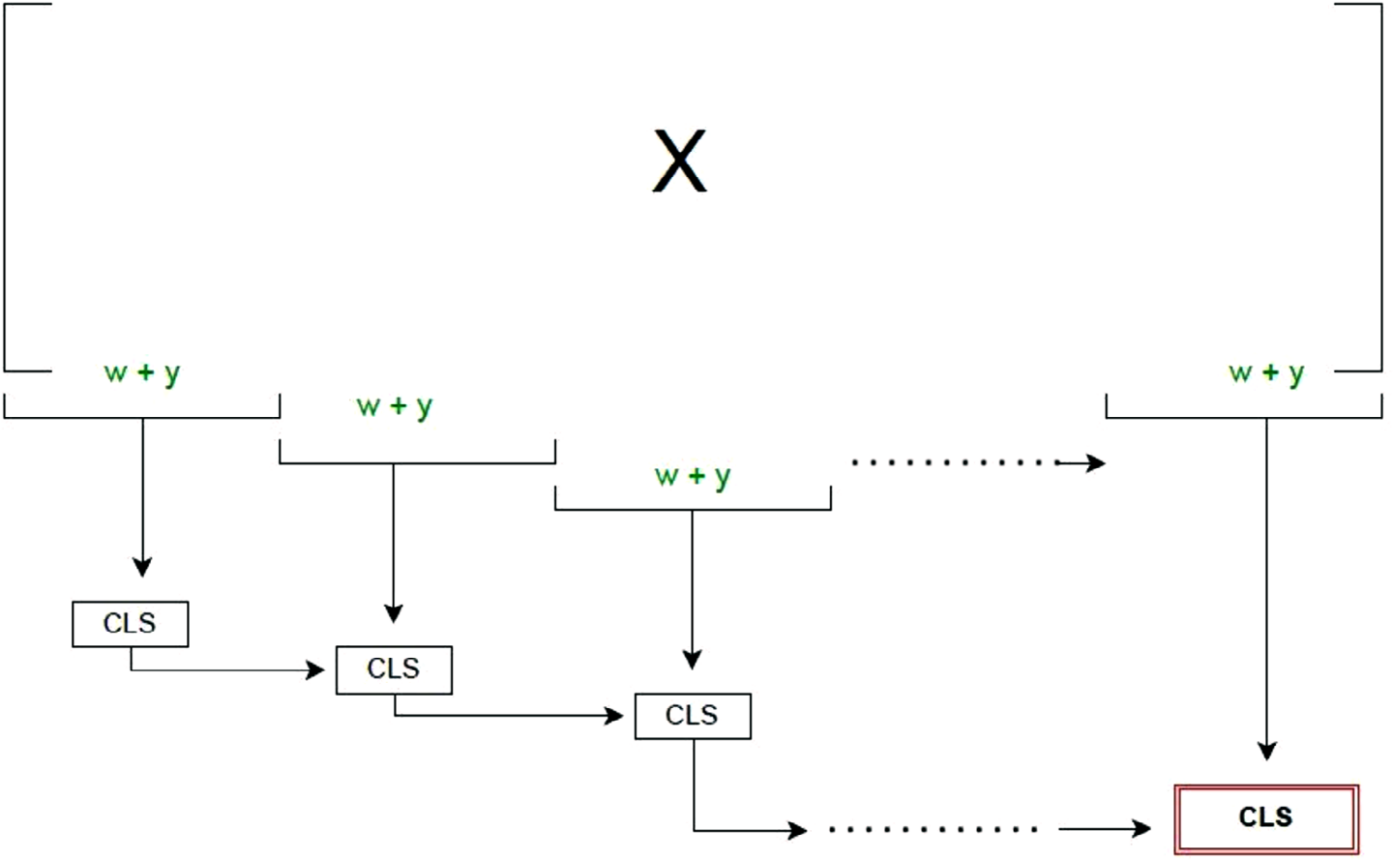
The Sliding Window approach for $X\in R^{n\times p}$. For one window, we consider a submatrix comprising w variables out of p and attach the response y to it. We slide through the whole matrix and calculate the cross leverage scores, respectively. At the end, we have a final set of cross leverage scores for all variables.

We first introduce two new approaches, which share the concept of a *moving* window. For both, we consider the matrix X consisting of all variables (e.g., SNPs). In the *Sliding Window approach*, as illustrated in Figure 3, we iterate through this matrix with a window consisting of w consecutive variables at a time and add the response y, respectively. We consider the submatrix starting at some index j for each window ${\tilde{X}}^{j,j+w-1}={\left[X_{\cdot ,j:(j+w-1)},y\right]}^{T}$. If the window size is small enough, we can efficiently calculate the QR‐decomposition and determine the CLS in the standard way described above. We start with the first window $X^{1,w}$ comprising the variables 1 up to w plus the response y and store their corresponding scores. Then, we move the window w variables forward and repeat this until we reach the end of the matrix X, where we calculate the final set of scores. The window size should be kept as constant as possible as is common in other data stream algorithms (e.g., Geppert et al. 2020; Vitter 1985). Therefore, w should be (roughly) a divisor of p. In a simulation study, we found that the size of w does not have a large impact on the result (see Figure A7 in Appendix A.2). The results indicate that w should be at least of order Ω(nlogn) to avoid rank‐deficient submatrices, which is in line with our previous discussion. Above this value, the gain of using larger w declines and becomes negligible. It should thus be chosen as large as possible but small enough to ensure that we can perform the calculations on submatrices efficiently. However, we found a more critical trade‐off in computation time between computing many small matrices or few large matrices, which will be discussed in detail in Section 7.

In the *Random Window approach*, the selection of variables in each window is chosen uniformly at random. For each window, we sample w out of p variables and add the response y, respectively. We repeat this a number of R times, but the algorithm also stops when every variable was chosen at least once before R steps. To ensure that every variable is chosen at least once, R should chose sufficiently large. If only one variable is chosen in each step, this can be quantified as R=Θ(plogp) by the coupon collector's theorem (Erdős and Rényi 1961). If the window size w is increased, we take a sample of w variables without replacement at a time that only increases the inclusion probability of each variable. Therefore, if this is repeated independently $R=O(\frac{p}{w}\log p)$ times, the resulting sample includes all variables with good probability. This will be discussed in more detail in Section 7. Analogously to previous considerations, w should be at least in Ω(nlogn) to avoid rank‐deficient submatrices. In Section 7, we discuss the parameter choice for the different approaches.

**FIGURE A2:**
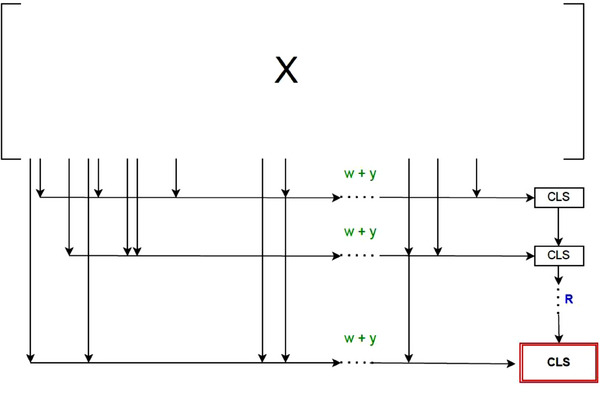
The Random Window approach for $X\in R^{n\times p}$. For each window, we consider a random submatrix comprising w out of p variables and attach the response y. We then calculate the cross leverage scores for the submatrix. We repeat this R times to get a final set cross leverage score to the sampled variables.

Each individual window is sampled from the set of variables without replacement, but variables can be sampled repeatedly in different windows. We always store the corresponding scores to the variables for every window. When one variable is chosen repeatedly, we compare the new score to the previous and store only the largest. Hereby, we try to avoid a possibly bad influence of the Random Window selection on the estimates of the CLS. The Random Window approach is illustrated in Figure A2 in Appendix A.2.

The advantage of these approaches is that they enable calculating CLS in scenarios where the direct calculations fail because there are too many variables to compute the QR‐decomposition as a whole. In genetic application, our data are often so large that we cannot even read it into the main memory of the computing environment such as R. Since our two approaches consider only small windows of the data at once, we only need to store and process the subset of data that we need for the current window. So, we mainly save a lot of memory by calculating the scores in that way, but indirectly also running time, since the two window approaches avoid swapping between the fast internal and slow external memory. However, one limitation of both approaches is that there are no theoretical guarantees available on the quality of the estimates they yield for the CLS. Therefore, we additionally consider a third approximation approach that provides rigorous theoretical guarantees.

### 3.3 Sketching Approach

We would like to calculate the CLS as in Equation (3), but we need to avoid the costly QR‐decomposition of the huge matrix $\tilde{X}$, which might even become intractable beyond some amount of data. The idea is to construct a significantly smaller “random sketch” of the input matrix of which we can calculate the QR‐decomposition. We therefore project the columns of $\tilde{X}$ to a lower dimensional subspace by multiplying a properly chosen sketching matrix $\Pi \in R^{r\times \tilde{p}}$ with $\tilde{X}\in R^{\tilde{p}\times n}$:

(4) $${\tilde{X}}_{★}=\Pi \tilde{X}\in R^{r\times n}.$$

The *Sketching approach* is a method to approximate the CLS by means of sketching (Drineas et al. 2012), which is a common data reduction tool for the design of algorithms for large data, distributed data, and data streams (Munteanu 2023). This technique allows us to approximate the CLS of arbitrarily large data within an arbitrary precision parameterized by ε>0. Our idea is transferring the idea of data reduction based on random projections and subsampling from reducing observations to selecting variables. To this end, we consider the transposed data matrix $\tilde{X}={\left[X,y\right]}^{T}\in R^{\tilde{p}\times n}$ again with p≫n and $\tilde{p}=p+1$. We use a sketching matrix Π that is a further development of a Clarkson–Woodruff embedding (Clarkson and Woodruff 2017). Π is a sparse matrix, whose nonzero entries are {−1,1}. The number of nonzero entries per column is fixed, but depends on the target dimension of the embedding. The original Clarkson–Woodruff embedding attains the sparsest possible structure, involving only one single nonzero entry per column. We give a simple example of such an embedding in Equation (5) for reducing from five to three dimensions.^1^

(5) $$\left(\begin{array}{ccccc} 0 & 0 & 1 & -1 & 0\\ -1 & 0 & 0 & 0 & 0\\ 0 & 1 & 0 & 0 & 1 \end{array}\right)\cdot \left(\begin{array}{c} x_{1}\\ x_{2}\\ x_{3}\\ x_{4}\\ x_{5} \end{array}\right)=\left(\begin{array}{c} x_{3}-x_{4}\\ -x_{1}\\ x_{2}+x_{5} \end{array}\right).$$

In general, there is a trade‐off between the sparsity and the target dimension. The target dimension (r in Equation (4)) of the original Clarkson–Woodruff embedding is $r=\Theta (n^{2})$. In our experiments, using oblivious subspace embeddings by Cohen (2016), we can sketch $\tilde{X}$ from $\tilde{p}$ down to only $r=O(\frac{n\log n}{\varepsilon^{2}})$ dimensions at the cost of a less sparse embedding with O(logn) nonzeros per column. We note that the most recent variant by Chenakkod et al. (2024) achieves even optimal size $r=O(\frac{n}{\varepsilon^{2}})$ with slightly worse sparsity of $O(\log^{4}n)$ nonzeros per column. Despite these differences between sketching techniques, the main property, we are interested in the scope of this paper, is the approximation error bound depending on ε, which remains the same for all variants. We thus refer the interested reader to the following related literature for further comparison between different sketching techniques.

Other dense sketching approaches such as ε‐JLT (Johnson and Lindenstrauss 1984), the Rademacher sketch Clarkson and Woodruff (2009), and the Subsampled Randomized Hadamard Transform (Ailon and Liberty 2009) are possible alternatives, which allow for different parameterizations in the trade‐off between the time needed to multiply the sketch and the required number of rows. We refer to Geppert et al. (2017) for experimental comparisons and overview. However, as pointed out previously, these approaches neither achieve a better accuracy nor a lower target dimension nor faster running time than the sparse Cohen–Sketch. We thus focus on the Cohen–Sketch (Cohen 2016) in our presentation and experiments.

From ${\tilde{X}}_{★}\in R^{r\times n}$ in Equation (4), we can easily determine the QR‐decomposition ${\tilde{X}}_{★}=Q_{★}R_{★}$ and calculate $R_{★}^{-1}$ in time and space independent of $\tilde{p}$. Now we can determine a matrix Ω as an approximation of the matrix Q from the original QR‐decomposition $\tilde{X}=QR$:

$$\Omega =\tilde{X}R_{★}^{-1}\in R^{\tilde{p}\times n}.$$

Finally, we can compute the approximation of the CLS of every single variable with the $\tilde{p}\text{th}$ variable (the attached response) by calculating the dot product of the respective rows of Ω:

$${\hat{c}}_{i\tilde{p}}=\left\langle \Omega_{i\cdot },\Omega_{\tilde{p}\cdot }\right\rangle ,\;i\in \left\{1,\text{…},p\right\}.$$

The whole procedure is summarized in Algorithm 1. The output of the algorithm satisfies the following guarantee (cf. Drineas et al. 2012, Lemma 5) with respect to the original CLS $c_{i\tilde{p}}$:

(6) $$\forall i\in \left\{1,\text{…},p\right\}:|c_{i\tilde{p}}-{\hat{c}}_{i\tilde{p}}|\leq \varepsilon ∥Q_{i\cdot }{∥}_{2}{∥Q_{\tilde{p}\cdot }∥}_{2}\leq \varepsilon .$$

ALGORITHM 1 **Approximation of the CLS of $\tilde{X}$.**

**Table:** 

**Input:** $\tilde{X}\in R^{\tilde{p}\times n}$ ($\tilde{p}=p+1$)	
**Output:** ${\hat{c}}_{i\tilde{p}},i\in \left\{1\text{…},p\right\}$	
1:	Project $\tilde{X}$ to a lower r‐dimensional subspace to obtain ${\tilde{X}}_{★}=\Pi \tilde{X}\in R^{r\times n}$ using, e.g., $r=\frac{n\cdot \log n}{\varepsilon^{2}}$ (Cohen 2016)
2:	Compute the QR‐decomposition ${\tilde{X}}_{★}=Q_{★}R_{★}$
3:	Compute $\Omega =\tilde{X}R_{★}^{-1}$ where $\Omega \in R^{\tilde{p}\times n}$
4:	Compute the CLS: ${\hat{c}}_{i\tilde{p}}=\left\langle \Omega_{i\cdot },\Omega_{\tilde{p}\cdot }\right\rangle$

See Drineas et al. (2012) for details. In particular, the guarantee given by Equation (6) implies that the large CLSs are well preserved. We remark that the largest entries of the CLS vector can be approximated within strictly lower dimensions than the original least squares problem Mai et al. (2023), which implies further computational benefits.

## 4 Data

### 4.1 Simulated Data

We simulate two different scenarios of SNP data, where we distinguish between three different genotypes: homozygous referent, homozygous variant, and heterozygous variant. These are usually encoded using the values {0,1,2} and the last two indicate the presence of an SNP. In the first scenario $S_{1}$, we consider one two‐way interaction and p−2 noisy variables.

$$S_{1}=\left({\mathrm{SNP}}_{1}\wedge {\mathrm{SNP}}_{2}\right).$$

For the number of variables, we choose p∈{2000,20000,200000,2000000}. We always fix n=120 observations and a binary outcome y∈{0,1}. We simulate 1000 independent data sets for p∈{2000,20000} and 100 data sets for p∈{200000,2000000}. We use the function simulateSNPglm from the R package scrime (Schwender 2018) to simulate the SNP data. All data are generated from the model

$$\begin{array}{cc} Y & ∼\mathrm{Bernoulli}(\mathrm{pred})\\ \mathrm{pred} & =\frac{1}{1+\exp(-\mathrm{lin}.\mathrm{pred})}, \end{array}$$

where $\mathrm{lin}.\mathrm{pred}=\beta_{0}+\beta_{1}M_{1}$, and $M_{1}=\left({\mathrm{SNP}}_{1}\wedge {\mathrm{SNP}}_{2}\right)$. Further, we choose $\beta_{0}=\log\left(\frac{0.3}{1-0.3}\right)$ and $\beta_{1}=\log\left(50\right)$. These values for β are chosen such that the specified interactions have a relatively large effect on the target variable. So, the probability that y=1 is 0.3 if $M_{1}$ is FALSE and roughly 0.95 if $M_{1}$ is TRUE. For all SNPs, we draw the minor allele frequency from a uniform distribution on the interval $\left[0.15,0.45\right]$. To validate how the approaches can deal with more complex interactions, we design a second scenario

$$S_{2}=\left({\mathrm{SNP}}_{1}\wedge {\mathrm{SNP}}_{2}\right)\vee \left({\mathrm{SNP}}_{3}\wedge {\mathrm{SNP}}_{4}\right).$$

For $S_{2}$, we choose $\mathrm{lin}.\mathrm{pred}=\beta_{0}+\beta_{1}M_{1}+\beta_{2}M_{2}$ with $M_{1}=\left({\mathrm{SNP}}_{1}\wedge {\mathrm{SNP}}_{2}\right)$, $M_{2}=\left({\mathrm{SNP}}_{3}\wedge {\mathrm{SNP}}_{4}\right)$, and $\beta_{0}=\log\left(\frac{0.3}{1-0.3}\right)$ and $\beta_{1}=\beta_{2}=\log\left(50\right)$.

All simulated data are available at Zenodo (Teschke 2024).^2^ The code is available on GitHub.^3^

### 4.2 Real Data

We also consider a small real data application, for which we use the HapMap data set taken from the R package SNPassoc (Moreno, Gonzalez, and Pelegri 2022). This data set consists of 9307 SNPs that belong to 22 chromosomes from the HapMap project (Thorisson et al. 2005) of the National Human Genome Research Institute. Additionally, the data contain information on the individuals' origin. We distinguish between European (CEU) and Yoruba (YRI) to encode the binary response. We note that the HapMap data set is rather small. Nevertheless, we decided to use it because we are primarily interested in a comparison between the approximation and the original, nonapproximated CLS method. For conducting the latter analysis, the data must necessarily have a small and tractable dimension p. Furthermore, our choice of the well‐researched HapMap data set is a baseline reference and thus allows comparisons to other related work.

## 5 Results of Simulation Study

All calculations were performed using R version R 4.3.0 (R Core Team 2023). In the following, we focus on the two simulated scenarios comprising a single two‐way interaction and two two‐way interactions, respectively. Hereby, we address the problem that arises when there is so much data to process, that the computations become too expensive and we can no longer calculate the CLS in the conventional way. To evaluate how well the approaches work, we count how many of the important variables we find on average when we choose the q variables with the largest CLS out of different dimensions p. As discussed above, we set q=⌈nlogn⌉=575 with n=120. For the different approaches, we choose the parameter settings summarized in Table 1. The specific choices are evaluated and discussed later in Section 7.

**TABLE 1:** This table shows the values we have chosen for the parameters of the different methods. The choice of values is discussed in Section 7. Unsuitable parameters are highlighted in bold.

	Random window		Sliding window	Sketching
p	w	R	w	ε
2 000	200	200	200	ε∈{0.5,0.2,0.1}
20 000	2 000	500	2 000	ε∈{0.5,0.2,0.1}
200 000	2 000	1 000	2 000	ε∈{0.5,0.2,0.1}
2 000 000	2 000	2 000	2 000	ε∈{0.5,0.2,0.1}

In the sketching approach, we reduce the dimension from p to $r=\lceil \frac{n\log n}{\varepsilon^{2}}\rceil$. It holds that r∈{2300,14375,57500} for n=120 and ε∈{0.5,0.2,0.1}, respectively. Note that some combinations of ε and p are not meaningful, since for very small ε and small values of p, we would increase the dimension p, in which case it is advisable to preserve the initial dimension. Nevertheless, we will use all ε for all p in our evaluation. For saving running time in the Random Window approach, we use optimized values $R≲\frac{p}{w}\log p$, that is, lower than discussed in Sections 3.2 and 7. For comparing the performance of our new approaches to standard methods, we calculate the correlations of each variable with the response and select the q variables attaining the largest correlations. Additionally, we compare to uniform sampling as a standard baseline.

**FIGURE 4:**
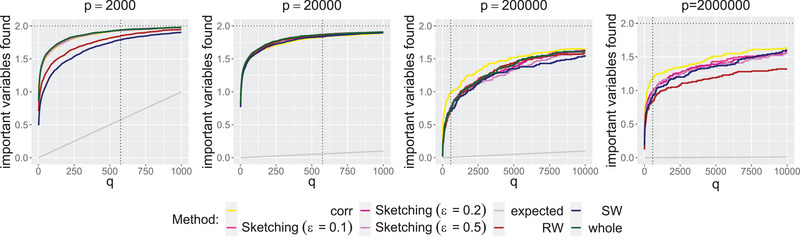
Important variables out of two important interacting variables (horizontal dotted line) found on average if we select the variables with the q largest CLS. At q=575, there is a vertical dotted line. We distinguish between the approaches by color. The gray line shows what we would expect if we select q variables uniformly at random.

In Scenario 1 (see Table A1 in Appendix A.2), all methods find on median average both important variables for p=2000 and p=20000. For p=200000 and p=2000000, we still find one out of two important variables on median average for all methods, whereby the CLS can only be calculated with approximation methods for p=2000000. The choice of the median is reasonable because the median is a robust measure of location and the number of variables found is dependent on the data set. To get a better comparison of the methods, we also consider the nonrobust mean. In Figure 4, we plot the mean number of important variables (out of two) found for different values of q and p across the various approaches.

We see that also for smaller values of q, we find on average almost two out of two important variables for all approaches for p=2000 and p=20000. For an increasing q, the number of variables found on average increases rapidly in the beginning and flattens out later, but we see that a smaller value of q would also suffice in practice. All methods are working well and the approximations differ only slightly. The gray line shows what we would expect if we select q variables uniformly at random. We see that this is consistently outperformed. For higher dimensions p=200000 and p=2000000, we see a similar pattern, but it should be noted that the plot includes considerably larger values of q on the horizontal axis here. Again, all approaches perform similarly well. However, on average, we do not find both important variables this time for the recommended q, but again, we clearly outperform the expected value of variables chosen uniformly at random (gray line). It seems that correlations work best in this case, but as we have seen in the toy example in Section 2, it cannot be relied upon in general. The plot indicates that we should choose a larger q for very large p.

**FIGURE 5:**
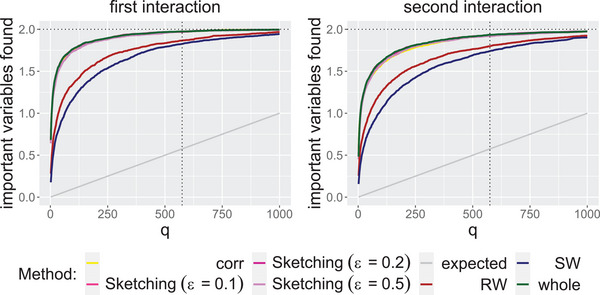
Important variables out of two (horizontal dotted line) found on average if we select the variables with the q largest CLS for the single two‐way interactions in the second scenario with p=2000. At q=575, there is a vertical dotted line. We distinguish between the approaches by color. Left: results for the first interaction. Right: results for the second interaction.

In the more complex Scenario 2, we first investigate how many out of the four important variables we find on median average (see Table A2 in Appendix A.2). We still find all important variables when selecting the variables with the largest CLS. This holds for both, the regular calculation of the CLS, and for the sketching methods, but also for the baseline using correlations. For larger p, we do not find all important variables any more, but still 3/4 for p=20000, and at least 1/4 for p=200000 and p=2000000 for all methods. In this case, it becomes interesting how often we find the respective complete interactions. Note that if we find only two important variables in total, it makes a difference whether we find a complete interaction or whether we find only one variable out of each interaction. For this purpose, we show how many variables we find on average for different values of q and different approaches for each of the two interactions separately. In Figure 5, we plot the curves for p=2000.

**FIGURE A3:**
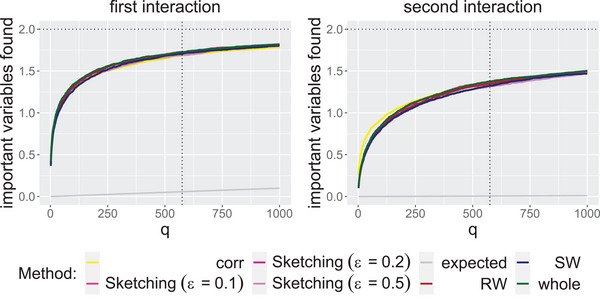
Important variables out of two (horizontal dotted line) found on average if we choose the variables with the q largest CLS for the single two‐way interactions in the second scenario with p=20000. At q=575, there is a vertical dotted line. We distinguish between the approaches by color. On the left side, we plotted the results for the first interaction and on the right side for the second.

**FIGURE A4:**
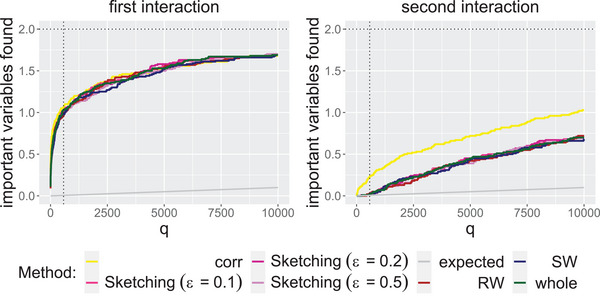
Important variables out of two (horizontal dotted line) found on average if we choose the variables with the q largest CLS for the single two‐way interactions in the second scenario with p=200000. At q=575, there is a vertical dotted line. We distinguish between the approaches by color. Left: results for the first interaction. Right: results for the second interaction.

**FIGURE A5:**
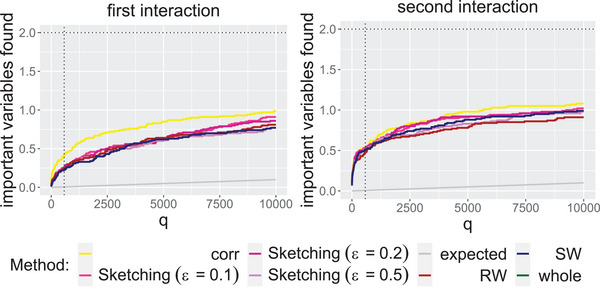
Important variables out of two (the vertical dotted line) found on average if we choose the variables with the q largest CLS for the single two‐way interactions in the second scenario with p=2000000. At q=575, there is a horizontal dotted line. We distinguish between the approaches by color. On the left side, we plotted the results for the first interaction and on the right side for the second.

The case p=20000 can be found in Figure A3 in Appendix A.2. As can be seen from Table A2 in Appendix A.2, both interactions for p=2000 are very likely to be found for q=575. However, it is remarkable how well the first interaction is found even for a smaller q. Even for p=20000, we found on average nearly all the important variables from the first interaction. For p=2000 as well as for p=20000, the first interaction is detected slightly more often for smaller values of q. The plots for larger values of p can be found in Figure A4 and Figure A5 in Appendix A.2. Also for p=200000, we see that the first interaction was found in more cases. For p=2000000, the two interactions are found roughly equally often. Again, it should be noted that the plot includes considerably larger values of q on the horizontal axis. It can be seen, however, that for smaller q, significantly fewer of the important variables are found here. For increasing p, q should also be increased, if necessary. Nevertheless, a selection with CLS outperforms a uniform random selection. Again, it can be stated that all methods, including the approximation methods, achieve similar results.

## 6 Real Data Application and Predictive Performance

In this section, we investigate how the variable selection using CLS affects predictive performance. To this end, we consider Random Forests (Breiman 2001) for the HapMap data, and logic regression (Ruczinski, Kooperberg, and LeBlanc 2003) for another simulated data set. The simulated data set is generated analogously to scenario $S_{1}$ from the previous section, but we consider only small values of p, so that we can apply logic regression to the full data for the sake of comparison. Our aim is to investigate whether we can improve the predictive performance by applying the variable selection methods instead of considering the whole data set.

### 6.1 Random Forests on HapMap Data

The data consists of p=7648 SNPs with a binary response and n=120 observations in total. We use Random Forests here to handle the full data with such a “large” value of p, which would not be possible using logic regression. We use the R‐package ranger (Wright and Ziegler 2017), with default parameter settings. To ensure that the measured effect on the performance comes from the specific choice of variable selection methods, and is not only a consequence of the mere variable reduction, we also consider the performance gain using a uniform random sample of variables as a baseline we would like to improve upon.

We perform the variable selection on a training data set on which we also train the Random Forest (using only the selected variables) and then test the predictive performance on the remaining test data. The fraction of training data is chosen to be 2/3, while the remaining 1/3 fraction represents the test data. Since we have n=120 observations, this means that 80 observations are used for training and 40 for testing.

Note that for the whole data set without preselection, we already have a prediction error of 0%. Thus, we choose a small value of q=10. We want to see what happens when we select q=10 random variables compared to a selection of q=10 variables by the largest CLS or correlations, respectively. We see (Table A3 in Appendix A.2) that using a uniform random sample, the prediction error increases to a nonzero error rate of 12.5%. On the other hand, the prediction error after variable selection according to the CLS remains at 0%, This supports that *meaningful* variables were selected by CLS (and correlations).

### 6.2 Logic Regression on Simulated Data

To consider the predictive performance with logic regression, we need a data set with considerably smaller p. We simulate again 1000 independent data sets with one two‐way interaction in the same way as for simulation scenario $S_{1}$, but only for p=500. Analogously to the HapMap data, we consider the predictive performance comparing no variable selection, variable selection with CLS or correlations, and a uniform random sample of variables. We split the data into test and training set and select q=25 variables based on the training data. For the calculations, we use the R‐package logicDT (Lau 2023). In Table A4 in Appendix A.2, we summarize the median prediction errors for all methods.

We see that the variable selection with CLS and correlations yield a prediction error of 35%. According to the t‐test, both outperform the model without variable selection significantly, which have a prediction error of 37.5%. Again, a selection by uniform sample has a much worse prediction error of 47.5%. In all cases, the prediction quality with logic regression is quite low,^4^ but we have shown that it improves significantly by using variable selection with CLS. And even in better performing settings such as Random Forests on the HapMap data, variable selection with CLS is useful.

**FIGURE 6:**
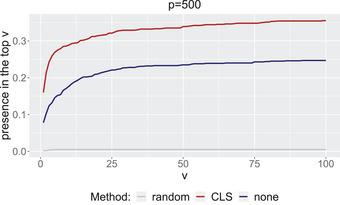
Presence of interaction between $X_{1}$ and $X_{2}$ in the top v important variables in the logic regression model: without variable selection (blue line), selection by the q largest CLS (red line), and uniform random selection of q variables (gray line).

Finally, we are interested in the variable importance measures (VIMs) of the logic terms identified by the logic regression model (Schwender and Ickstadt 2008). We obtain VIMs from the model for various main effects as well as for interaction effects of different orders. In the logicDT package, we use the parameter setting vim_type = “logic” and ave = “before”, which compares the average performance over fixing one Boolean variable to {0,1}, respectively. We refer to Lau (2023) for details on available parameter settings. We would like to investigate whether we can obtain better results for the VIMs after performing a variable selection using CLS. To this end, we select the q=25 variables with the largest CLS on the training data, and calculate the VIMs on the test data. We also consider the VIMs using a uniform random sample of q=25 variables for the sake of comparison. Additionally, we calculate the VIMs in the same way without any preselection. We count how often the important interaction between $X_{1}$ and $X_{2}$ is among the top v most important variables, respectively, logic terms over 1000 data sets in terms of the calculated VIM. In Figure 6, it is shown that applying a variable selection method beforehand results in a considerable improvement, and we find the relevant interaction more often in the top v of logical terms measured by the variable importance.

## 7 Discussion and Conclusion

When we consider more complex scenarios, such as, for instancethree‐ or four‐way interactions, identifying all variables of the respective interaction becomes more difficult. However, if p is not too large, our methods still work. In case of p=2000, we still find all important variables for the three‐way interaction and at least two out four important variables for the four‐way interaction on median average, when selecting these variables with the q=575 largest CLS. For larger p, this becomes more difficult and the results deteriorate. Known findings in high‐dimensional statistics suggest that a log(p) dependence might be necessary (Amini and Wainwright 2009; Mai et al. 2023).

**FIGURE 7:**
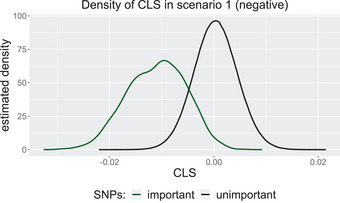
Kernel density estimates of the CLS of the important (green) and unimportant (black) variables in the scenario where an existing two‐way interaction has an negative influence on the response (p=2000).

So far, we have only considered scenarios where an interaction of SNPs has a positive effect on the response. In this case, it makes sense to select the variables attaining the largest CLS. However, if we consider scenarios in which the absence of an SNP interaction favors a disease (y=1), then the corresponding important variables have a largely *negative* CLS. This is illustrated in Figure 7 (in contrast to Figure 1).

**FIGURE A6:**
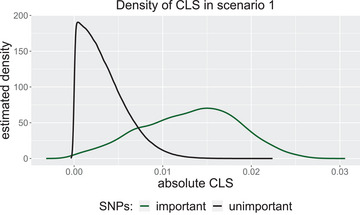
Kernel density estimates of the absolute CLS of the important (green) and unimportant (black) variables in the scenario $S_{1}$ and p=2000.

So, if we do not know anything about the data, it is recommended to select the variables attaining the largest absolute values of their CLS. Nevertheless, also in scenario $S_{1}$, the absolute scores could still be used to distinguish the important from the unimportant variables, see Figure A6 in Appendix A.2.

In our paper, we select exactly the q variables with the largest (absolute) CLS. But, in the presence of outliers and to preserve the whole space spanned by the data, it was recommended to sample q variables weighted by their CLS including a small fraction of standard leverage scores (Parry et al. 2021) instead of greedily selecting the largest scores. However, in this paper, we used the CLS as a measure of importance of individual variables that justifies selecting the variables without sampling.

**FIGURE A7:**
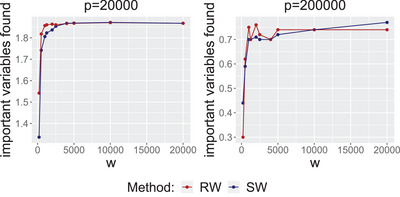
Number of important variables of two found on average if we choose the variables with the q=nlog(n) largest CLS for the single two‐way interactions in the second scenario with p=20000 and p=200000 for different window sizes w. Different approaches are indicated by color.

Another point to discuss is the choice of parameters for the two window‐based approaches. For the following analyses, we considered the scenario $S_{1}$ with one two‐way interaction and p=20000. How should we choose the window width w in the Sliding Window and Random Window approaches? First, the smaller we choose the window size w, the faster we can calculate the QR‐decomposition, but this also increases the number of QR‐decompositions to be computed. Our analyses have shown that w should not be chosen too small, in particular, w≳nlogn, but beyond some threshold, its choice does not make a big impact on the performance. Figure A7 in Appendix A.2 illustrates this. However, the intuitive reasoning holds that the larger w is, the better interactions can be detected on average. This applies to both window‐based approaches. In the Random Window approach, a larger value of w implies that it takes less iterations until each variable is drawn at least once, but the individual computations for the QR‐decompositions in each iteration take longer. Similar considerations apply to the parameter R, which determines how many windows we consider in the Random Window approach. A large number provides an increased running time, but no performance improvement, as long as R is chosen large enough so that each variable is selected at least once (with high probability), which is the case for $R\approx \frac{p}{w}\log p$. In Table 2, we show the respective values for R. Nevertheless, for the specification of R and w, it is recommended to consider the trade‐off between how fast we want to calculate the scores and how large we (have to) choose the individual submatrices. In the sketching approach, we can vary the parameter ε, which determines the accuracy guarantee of the approximation of the CLS and provides a trade‐off to the size of the sketched matrix that again has a crucial impact on the time and space required to perform the QR‐decomposition.

**TABLE 2:** This table shows values for $R\approx \frac{p}{w}\log p$ in the Random Window approach.

p	w	R
2 000	200	76
20 000	2 000	99
200 000	2 000	1221
2 000 000	2 000	14,509

In conclusion, we showed that the CLSs are appropriate for distinguishing important from unimportant variables, even if they only have an indirect influence on the response through variable interactions. The advantage of CLS is that they can be calculated directly from the data matrix and that it is not necessary to consider the vast space of every possible variable interaction. Especially for higher order interactions and more complex scenarios, this brings massive advantages compared to standard methods. Our approximations to CLS work reliably and interaction effects can be identified even for very large p in the order of millions, where previous methods face severe computational limitations. With the selected variables via the CLS, conventional downstream regression and classification analyses can be applied, which would fail on the original high‐dimensional input. The previous selection using CLS has a positive influence on their predictive performance and calculation of VIMs. Not to mention that many methods would simply not be applicable without a prior variable selection respectively reduction in the presence of massive amounts of data commonly considered in genome‐wide analyses. Even though we have limited this paper to SNP data and binary response, the methods presented here can also be applied to arbitrary real‐valued data types.

## Conflicts of Interest

The authors have declared no conflict of interest.

### Open Research Badges

This article has earned an Open Data badge for making publicly available the digitally‐shareable data necessary to reproduce the reported results. The data is available in the Supporting Information section.

This article has earned an open data badge “**Reproducible Research**” for making publicly available the code necessary to reproduce the reported results. The results reported in this article could fully be reproduced.

## Supporting information

Supporting Information

## Data Availability

The data that support the findings of this study are openly available in Zenodo at https://doi.org/10.5281/zenodo.

## Footnotes

1 The example is taken from Nayebi, Munteanu, and Poloczek (2019).

2
https://doi.org/10.5281/zenodo.12742957

3
https://github.com/SvenTeschke/special_issue_CEN

4 Note that its main purpose is detecting interacting variables rather than making preditions.

## Appendix A

### A.1 Poof of Proposition 3.1

Let $\tilde{X}=\left[X,y\right]\in R^{n\times p+1}$, for p≫n, where X and thus also $\tilde{X}$ have full rank n. Consider the $\ell_{2}$ regression problem

(A1)
$$\min_{\beta \in R^{p}}{∥X\beta -y∥}_{2}^{2}\;.$$

Since p≫n the problem is underconstrained and therefore there exists a subspace $L\subset R^{p}$ such that any β∈L satisfies Xβ=y. Consequently, any β∈L constitutes an optimal solution where Equation (A1) equals 0. Now, consider the (thin) singular value decomposition (Golub and Van Loan 1996) $\tilde{X}=U\Sigma V^{T}$, where $U\in R^{n\times n}$ is orthonormal, $\Sigma \in R^{n\times n}$ is diagonal whose entries are the singular values $\sigma_{i}\left(\tilde{X}\right)>0$, for i∈[n], and $V^{T}\in R^{n\times p+1}$ has orthonormal rows. We further let $V^{T}=\left[V_{X}^{T},V_{y}^{T}\right]$, where $V_{X}^{T}$ carries the first p columns corresponding to the columns of X, while $V_{y}^{T}$ is the (p+1)st column that corresponds to y. With these decompositions in place, Equation (A1) can be rewritten as the minimization of

$$\begin{array}{c} {∥X\beta -y∥}_{2}^{2}={∥U\Sigma \;\left(V_{X}^{T}\beta -V_{y}^{T}\right)∥}_{2}^{2}\;. \end{array}$$

In particular, note that it implies

$${∥X\beta -y∥}_{2}^{2}=0\;⟺\;{∥V_{X}^{T}\beta -V_{y}^{T}∥}_{2}^{2}=0\;.$$

Next, we make use of the fact that a zero error solution can be obtained in closed form by means of the Moore–Penrose pseudoinverse (Ben‐Israel and Greville 2003), which for the RHS yields the (unique) least $\ell_{2}$‐norm solution

$$\begin{array}{c} \beta^{OLS}=V_{X}{\left(V_{X}^{T}V_{X}\right)}^{-1}V_{y}^{T}. \end{array}$$

If $V_{X}^{T}$ were orthonormal, this would simplify even further to the vector comprising the CLS as its coordinates, since in that case ${\left(V_{X}^{T}V_{X}\right)}^{-1}=I^{-1}=I$ and we have $c_{\cdot ,p+1}=V_{X}V_{y}^{T}$ by definition. However, $V_{X}$ is not quite orthonormal since it was obtained by removing one row vector $V_{y}$ from the orthonormal basis V. But since we removed only one row of small, bounded norm, we can argue that it is close to being orthonormal in what follows. Interpreting the matrix inner product as a sum of rank‐1 tensors, we have that

(A2)
$$\begin{array}{cc} ∥V^{T}V-V_{X}^{T}V_{X}{∥}_{2} & ={\left\|\sum_{i=1}^{p+1}V_{i}^{T}V_{i}-\sum_{i=1}^{p}V_{i}^{T}V_{i}\right\|}_{2}\\ & =∥V_{y}^{T}V_{y}{∥}_{2}={∥V_{y}∥}_{2}^{2}. \end{array}$$

We now assume that after normalization of the space, the columns are well aligned with the normalized response vector. Note that this is natural to assume since otherwise the regressors would be (almost) orthogonal to the response, in which case a regression model would not make any sense at all. Formally, we require that for some data dependent constant η∈(0,1/2] the following holds

(A3)
$$\sum_{i=1}^{p}{\left(V_{i}\frac{V_{y}^{T}}{∥V_{y}{∥}_{2}}\right)}^{2}\geq \frac{1}{\eta }{∥V_{y}∥}_{2}^{2}\;.$$

Note that the threshold is independent of the data dimensions and is *not* required to grow with the numbers of observations or variables. We only require some *constant* amount of the projected information to be aligned with the column that belongs to the response variable. Now consider the standard leverage score of the response variable, which we define and bound from above as

$$\ell_{y}=\underset{u\in R^{n}\setminus \left\{0\right\}}{\sup}\frac{|V_{y}^{T}{u|}^{2}}{{∥Vu∥}_{2}^{2}}\overset{CSI}{\leq }\frac{∥V_{y}^{T}{∥}_{2}^{2}{∥u∥}_{2}^{2}}{{∥Vu∥}_{2}^{2}}={∥V_{y}∥}_{2}^{2},$$

where we used the Cauchy–Schwarz inequality (CSI). We further note that strict equality holds for the unit vector $u=V_{y}^{T}/{∥V_{y}^{T}∥}_{2}$. Consequently, it follows that

$$\begin{array}{cc} ∥V_{y}{∥}_{2}^{2} & =∥V_{y}^{T}{∥}_{2}^{2}{\left\|\frac{V_{y}^{T}}{∥V_{y}^{T}{∥}_{2}}\right\|}_{2}^{2}\;/\;{\left\|V\frac{V_{y}^{T}}{∥V_{y}^{T}{∥}_{2}}\right\|}_{2}^{2}\\ & =\frac{∥V_{y}^{T}{∥}_{2}^{2}}{{\left(V_{y}\frac{V_{y}^{T}}{∥V_{y}^{T}{∥}_{2}}\right)}^{2}+\sum_{i=1}^{p}{\left(V_{i}\frac{V_{y}^{T}}{∥V_{y}^{T}{∥}_{2}}\right)}^{2}}\\ & \overset{\left(A3\right)}{\leq }\frac{∥V_{y}{∥}_{2}^{2}}{∥V_{y}{∥}_{2}^{2}\left(1+\frac{1}{\eta }\right)}\leq \eta \;. \end{array}$$

Combining the previous calculations (A2) with our assumption (A3) thus implies that

(A4)
$$∥I-V_{X}^{T}V_{X}{∥}_{2}\leq \eta \;.$$

To get a bound on the $\beta^{OLS}$ estimator above, we need a similar bound for the inverse ${\left(V_{X}^{T}V_{X}\right)}^{-1}$ quantifying the amount by which it deviates from the identity ${\left(V^{T}V\right)}^{-1}=I$. To this end, consider the eigenvalue decomposition $V_{X}^{T}V_{X}=Q\Lambda Q^{T}$, where $Q\in R^{n\times n}$ is an orthonormal basis satisfying $Q=Q^{T}=Q^{-1}$ and thus $QQ^{T}=Q^{T}Q=I$, and $\Lambda \in R^{n\times n}$ is a diagonal matrix whose entries are the eigenvalues $\lambda_{i}=\sigma_{i}^{2}\left(V_{X}\right)>0$, for i∈[n]. We have

$$\begin{array}{cc} ∥{\left(V^{T}V\right)}^{-1}-{\left(V_{X}^{T}V_{X}\right)}^{-1}{∥}_{2} & =∥I-{\left(V_{X}^{T}V_{X}\right)}^{-1}{∥}_{2}\\ & =∥QQ^{T}-{\left(Q\Lambda Q^{T}\right)}^{-1}{∥}_{2}\\ & =∥QQ^{T}-Q\Lambda^{-1}Q^{T}{∥}_{2}\\ & =∥I-\Lambda^{-1}{∥}_{2}\\ & =\max_{i\in [n]}\left|1-\frac{1}{\lambda_{i}}\right|=\max_{i\in [n]}\left|\frac{1-\lambda_{i}}{\lambda_{i}}\right|\\ & \leq \frac{\max_{i\in [n]}\left|1-\sigma_{i}^{2}\left(V_{X}\right)\right|}{\min_{i\in [n]}\sigma_{i}^{2}\left(V_{X}\right)}\overset{\left(A4\right)}{\leq }\frac{\eta }{1-\eta }\leq 2\eta . \end{array}$$

Then, it follows that

$$\begin{array}{cc} \max_{i\in [p]}\left|\beta_{i}^{OLS}-c_{i,p+1}\right| & =∥V_{X}{\left(V_{X}^{T}V_{X}\right)}^{-1}V_{y}^{T}-V_{X}V_{y}^{T}{∥}_{\infty }\\ & =∥V_{X}{\left(V_{X}^{T}V_{X}\right)}^{-1}V_{y}^{T}-V_{X}{\left(V^{T}V\right)}^{-1}V_{y}^{T}{∥}_{\infty }\\ & \leq ∥V_{X}\left({\left(V_{X}^{T}V_{X}\right)}^{-1}-{\left(V^{T}V\right)}^{-1}\right)V_{y}^{T}{∥}_{2}\\ & \leq ∥V_{X}{∥}_{2}∥{\left(V_{X}^{T}V_{X}\right)}^{-1}-{\left(V^{T}V\right)}^{-1}{∥}_{2}{∥V_{y}^{T}∥}_{2}\\ & \leq 1\cdot 2\eta \cdot \sqrt{\eta }<\frac{3\eta }{2}, \end{array}$$

which implies that each $\mathrm{CLS}c_{i,p+1}$ for i∈[p] equals their corresponding parameter in the least $\ell_{2}$‐norm solution up to a small additive error bounded in terms of η.□

### A.2 Additional Figures and Tables

**TABLE A1:** This table shows how many important variables out of two we find with the different methods in median average in scenario 1 (one two‐way interaction) if q=575. “CLS” means to calculate the CLS for the whole matrix, and RW and SW stand for the Random Window or Sliding Window Approach. We consider different values for ε(0.5,0.2,0.1) in the Sketching approach and the correlation as baseline method. Note that the case p=2000000 is only feasible for the approximation methods, not for the exact CLS calculations, indicated by X.

Median		Window		Sketching ε=			
p	CLS	RW	SW	0.5	0.2	0.1	correlation
2000	2	2	2	2	2	2	2
20000	2	2	2	2	2	2	2
200000	1	1	1	1	1	1	1
2000000	X	1	1	1	1	1	1

**TABLE A2:** This table shows how many important variables out of two we find with the different methods in median average in scenario 2 (two 2‐way interactions) if q=575. “CLS” means to calculate the CLS for the whole matrix, and RW and SW stand for the Random Window or Sliding Window Approach. We consider different values for ε(0.5,0.2,0.1) in the sketching approach and the correlation as baseline method. Note that the case p=2,000,000 is only feasible for the approximation methods, not for the exact CLS calculations, indicated by X.

median		Window		Sketching ε=			
p	CLS	RW	SW	0.5	0.2	0.1	correlation
2000	4	4	4	4	4	4	4
20000	3	3	3	3	3	3	3
200000	1	1	1	1	1	1	1
2000000	X	1	1	1	1	1	1

**TABLE A3:** Prediction error on HapMap data with and without variable and random selection beforehand.

Method:	Without	Random	CLS	corr
Prediction error:	0%	12.5%	0%	0%

**TABLE A4:** Prediction error on scenario $S_{1}$ with p=500 with and without variable and random selection beforehand.

method:	Without	Random	CLS	corr
Prediction error:	37.5%	47.5%	35.0%	35.0%
